# The role of Small Intestinal Bacterial Overgrowth (SIBO) in Non-alcoholic Fatty Liver Disease (NAFLD) patients evaluated using Controlled Attenuation Parameter (CAP) Transient Elastography (TE): a tertiary referral center experience

**DOI:** 10.1186/s12876-019-0960-x

**Published:** 2019-03-20

**Authors:** Yoga Fitriakusumah, C. Rinaldi A. Lesmana, Winda Permata Bastian, Chyntia O. M. Jasirwan, Irsan Hasan, Marcellus Simadibrata, Juferdy Kurniawan, Andri Sanityoso Sulaiman, Rino A. Gani

**Affiliations:** 1 Department of Internal Medicine, Hepatobiliary Division, Dr. Cipto Mangunkusumo National General Hospital, Universitas Indonesia, Jakarta, Indonesia; 2 Digestive Disease & GI Oncology Center, Medistra Hospital, Jakarta, Indonesia; 3 Department of Internal Medicine, Gastroenterology Division, Dr. Cipto Mangunkusumo National General Hospital, Universitas Indonesia, Jakarta, Indonesia

**Keywords:** SIBO, NAFLD, Transient Elastography

## Abstract

**Background:**

Non-alcoholic fatty liver disease (NAFLD) is an emerging disease, where it can progress to non-alcoholic steatohepatitis (NASH) and lead to liver cirrhosis or liver cancer. Small intestinal bacterial overgrowth (SIBO) has been hypothesized to play an important role in NAFLD development and progression, however, there is still conflicting data about this phenomenon. Transient Elastography (TE) examination using controlled attenuation parameter (CAP) has been validated for liver disease progression assessment in NAFLD. It is non-invasive method and easy to perform in clinical practice. Therefore, we would like to know the role of SIBO in NAFLD and its possible impact on disease progression.

**Methods:**

A cross-sectional design study performed at outpatient’s Hepatobiliary clinic at tertiary referral university hospital in Jakarta. All recruited study subjects based on inclusions criteria underwent laboratory examination, transabdominal ultrasound examination, CAP-TE 502 (by Echosens, France), and glucose hydrogen breath test (GHBT) using portable hydrogen breath test apparatus (Gastro+™ Gastrolyzer by Bedfont Scientific Ltd). Stool sample examination was performed using RT-PCR.

**Results:**

This study recruited 160 subjects with median age of 58 (22–78) years and 108 (67.5%) of them are female. SIBO (65,5%), DM (70.8%), dyslipidemia (75.2%), obesity (76.6%), and metabolic syndrome (73%) were more prevalent in NAFLD than non-NAFLD population. Bivariate analysis showed no significant association between SIBO and NAFLD development (*p* = 0.191; PR 0.871; CI 95% [0.306–1.269]). SIBO was also not associated with significant hepatic steatosis (*p* = 0.951; PR = 0.951; CI 95% [0.452–2.239]) and fibrosis (*p* = 0.371; PR = 1.369; CI 95% [0.608–3.772]). However, the presence of central obesity has significantly associated with the presence of SIBO (*p* = 0.001; PR = 0.378; CI 95% [0.021–0.478]). Based on stool sample analysis from 60 NAFLD patients, there is a significant correlation using Spearmen test between the presence of Bacteroides and the stage of fibrosis (p .037). Further analysis between obese NAFLD patients and non-obese NAFLD patients showing that there is a significant decrease of Bifidobacteria (p .047) and Lactobacillus (p .038) in obese NAFLD patients and a tendency of increase Bacteroides in obese NAFLD patients (p .572).

**Conclusions:**

SIBO is not associated with NAFLD development and progression.

## Background

Non-alcoholic fatty liver disease (NAFLD) is an emerging disease not only in Western countries, but also in Asian countries. Non-alcoholic steatohepatitis (NASH) is an important phenotype of NAFLD which responsible to the liver disease progression and it is a complex disease because of multi-factorial metabolic conditions [1–4]. Recently, multiple hit theory is known as the primary pathogenesis of NAFLD, whereas small intestinal bacterial overgrowth (SIBO) has been hypothesized to have an important role in liver disease progression in NASH, however, there is still inconsistency between studies about the role of SIBO, especially its impact on management [5–7]. SIBO is a condition where excessive amount of gut microbiota presents in the small intestines [7]. Therefore, in this study we would like to explore more about the association of SIBO in NAFLD patients and possible impact on disease progression.

Liver disease progression is one of a challenging condition in clinical practice as it is not easy to assess the early changing of the liver architecture and it would need follow up liver biopsy. Currently, transient Elastography (TE) has been used for liver disease progression assessment in chronic hepatitis infection. The controlled attenuation parameter (CAP) innovation with TE examination showing reliable result to assess not only hepatic fibrosis but also the degree of hepatic steatosis based on CA*P* value [8, 9].

## Methods

A cross-sectional study was performed at outpatient’s Hepatobiliary clinic at tertiary referral university hospital in Jakarta. Inclusion criteria is patients who have ≥1 metabolic risk factor for NAFLD development (diabetes mellitus (DM), obesity, dyslipidemia) and does not have history of significant alcohol consumption (> 21 drinks per week in men and > 14 drinks per week in women over a 2-year period). Exclusion criteria are irritable bowel syndrome (IBS), hepatitis B (HBsAg positive or anti HBc positive in HBsAg negative patients) & C virus infection, autoimmune liver disease, drug induced liver injury, hepatocellular carcinoma, gastrointestinal malignancy, inflammatory bowel disease (IBD), and patients with history of gastrointestinal surgery. Patients with history of drug or supplements consumption which can influence liver steatosis condition such as vitamin E, pioglitazone, amiodarone, tamoxifen, and Ursodeoxycholic acid are also excluded. Obesity is defined when BMI > 25 Kg/m^2^ (Asia Pacific criteria), and central obesity is defined based on WHO criteria for Asian population (male waist circumference ≥ 90 cm and female waist circumference ≥ 80 cm). All subjects underwent laboratory examination, transabdominal ultrasound examination, CAP-TE 502 (by Echosens, France), and glucose hydrogen breath test (GHBT) using portable hydrogen breath test apparatus (Gastro+™ Gastrolyzer by Bedfont Scientific Ltd). Subjects underwent strict diets consisted of avoiding easily fermented food such as complex carbohydrate (fruit, vegetable and grains) the day before GHBT and fasted for 8–12 h before GHBT. Antibiotics stopped at least 4 weeks and also laxatives 1 week before GHBT. Subjects brushed their teeth and then gargle using Chlorhexidine 0.2% solution at least 2 h before GHBT. GHBT done by instructing the subjects to inhale as deep as possible and hold it for 15 s and then exhaled it to the GHBT device, measurements were taken every 15 min in 120 min’ test. SIBO diagnosed if there is an increase of hydrogen concentration ≥ 20 ppm from baseline within the first 120 min. NAFLD diagnosis is confirmed by transabdominal ultrasound criteria (liver to kidney echogenicity), whereas the degree of steatosis and fibrosis are diagnosed based on CAP-TE examination. Steatosis cut off as follows: 263–280.9 dB/m (S1); 281–282.9 dB/m (S2); ≥283 dB/m (S3) and steatosis > 280.9 dB/m defined as significant steatosis. Significant fibrosis (F2) cut off is ≥7.1 kPa and advanced fibrosis (F3-F4) cut off is ≥9.5 kPa [9]. The examinations were performed by well-trained internal medicine fellow supervised by junior consultant and confirmed by senior consultant.

Patients who are also agreed to undergo stool samples examination for real time PCR quantification for Bifidobacterium sp., Lactobacillus sp., and Bacteroides sp. were taken written consent. Data analysis was done using SPSS ver.20. Bivariate analysis was performed using chi square test and multivariate analysis was performed using logistic regression test. This study has been approved by IRB/Institutional Review Board (Medical Faculty Universitas Indonesia Ethical Committee) no. 899/UN2.F1/ETIK/2017.

### Sample size calculation

$$ n1=n2=\frac{{\left({Z}_{\alpha}\sqrt{2 PQ}+{Z}_{\beta}\sqrt{P1\ Q1+P2\ Q2}\right)}^2}{{\left(P1-P2\right)}^2} $$

n = Sample size

α = 0,05; Z_α_ = 1,96

β = 0,80; Z_β_ = 0,842

P_1_ = proportion of standard effect (literature)

P_2_ = proportion of research effect (by author)

P = ½ (P_1_ + P_2_)

Q = 1 – P; Q_1_ = 1-P_1_

Q_2_ = 1- P_2_

Sample size for cross-sectional study design two proportion independent group:

Table sample size estimation

For Logistic regression analysis:

$$ \mathrm{N}=\frac{\mathrm{Independentvariables}\times 10}{\mathrm{Prevalence}} $$

Minimum samples (4 × 10)/50% = 80 samples, with additional 10% then the result is 88 samples. With the two proportions become 176 samples. From minimum independent variable: 54 × 2 = 108 samples.

### Stool sample examination

Stool sample collection was done using sterile kit and placed in the sterile tube. Then it is stored at 2-8 °C before it was sent to the Microbiology for DNA extraction process with DNA stool kit (TIANamp, TIANGEN Biotech (Beijing) Co., Ltd.). Real time (RT) PCR is performed using forward primer and specific reverse for Bifidobacterium sp., Lactobacillus sp., and Staphylococcus sp. with Universal SYBR® Green supermix.

## Results

### Patient’s characteristics and metabolic factors prevalence

There are 160 subjects with the median age 58 (22–78) years old who can be recruited based on inclusions and exclusions criteria, which consist of 52 (32.5%) male and 108 (67.5%) female. NAFLD was diagnosed in 115 (71.9%) patients, whereas SIBO was found in 55 (34.4%) patients. Other metabolic factors such as obesity was found in 107 (66.9%) patients, type 2 diabetes mellitus (DM) in 120 (75%) patients, dyslipidemia in 129 (80.6%) patients, and central obesity in 149 (93.1%) patients. Significant liver fibrosis (F2-F4) was only found in 26 (22.6%) patients (Table 1).

**Table 1:** Baseline Characteristics

Variables	Total (*N* = 160)
Gender, n (%)	
Male	52 (32.5)
Female	108 (67.5)
Age (years), median (min-max)	58 (22–78)
NAFLD, n (%)	
Yes	115 (71.9)
No	45 (28.1)
SIBO, n (%)	
Yes	55 (34.4)
No	105 (65.6)
Obesity, n (%)	
Yes	107 (66.9)
No	53 (33.1)
DM, n (%)	
Yes	120 (75)
No	40 (25)
Dyslipidemia, n (%)	
Yes	129 (80.6)
No	31 (19.4)
Metabolic Syndrome, n (%)	
Yes	122 (76.3)
No	38 (23.8)
Central Obesity, n (%)	
Yes	149 (93.1)
No	11 (6.9)
Fibrosis Degree, n (%)	
F0-F1	89 (77.4)
F2-F4	26 (22.6)
Steatosis Degree, n (%)	
S0	41 (26.4)
S1	10 (6.4)
S2	4 (2.5)
S3	60 (38.7)
Significant Steatosis, n (%)	
Yes	71 (61.7)
No	44 (38.3)

*SIBO* Small Intestinal Bacterial Overgrowth, *NAFLD* Non-Alcoholic Fatty Liver Disease, *DM* Diabetes Mellitus, *PR* Prevalence Ratio, *CI* Confidence Interval

In NAFLD patients, the prevalence of metabolic factors is dominated by obesity (76.6%), followed by dyslipidemia (75.2%), central obesity (73.2%), metabolic syndrome (73%), and type 2 DM (70%). SIBO was found in 65.5% patients with NAFLD. This prevalence is higher than in non-NAFLD patients.

### Association between SIBO and NAFLD

Bivariate analysis showed no significant association between SIBO and NAFLD development (*p* = 0.191; PR 0.871; CI 95% [0.306–1.269]) (Table 2) All other metabolic factors (DM, dyslipidemia, obesity, and central obesity) were also not shown as independent risk factors for NAFLD development (Table 3).

**Table 2:** Bivariate Analysis Association between SIBO and NAFLD

Variables	NAFLD, n (%)		Total	*p* value	PR	CI 95%
	Yes	No				
SIBO, n (%)						
Yes	36 (65.5)	19 (34.5)	55 (100)	0.191	0.624	0.306–1.269
No	79 (75.2)	26 (24.8)	105 (100)			

*SIBO* Small Intestinal Bacterial Overgrowth, *NAFLD* Non-Alcoholic Fatty Liver Disease, *PR* Prevalence Ratio, *CI* Confidence Interval

**Table 3:** Bivariate Analysis Association between Comorbidities and NAFLD

Variables	NAFLD, n (%)		Total	*p* value	PR	CI 95%
	Yes	No				
DM, n (%)						
Yes	85 (70.8)	35 (29.2)	120 (100)	0.612	0.810	0.358–1.832
No	30 (75.0)	10 (25.0)	40 (100)			
Dyslipidemia, n (%)						
Yes	97 (75.2)	32 (24.8)	129 (100)	0.057	2.189	0.966–4.959
No	18 (58.1)	13 (41.9)	31 (100)			
Obesity, n (%)						
Yes	82 (76.6)	25 (23.4)	107 (100)	0.057	1.988	0.974–4.057
No	33 (62.3)	20 (37.7)	53 (100)			
Metabolic Syndrome, n (%)						
Yes	89 (73)	33 (27)	122 (100)	0.588	1.245	0.564–2.748
No	26 (68.4)	12 (31.6)	38 (100)			
Central Obesity, n (%)						
Yes	109 (73.2)	40 (26.8)	149 (100)	0.294	2.271	0.657–7.854
No	6 (54.5)	5 (45.5)	11 (100)			

*DM* Diabetes Mellitus, *NAFLD* Non-Alcoholic Fatty Liver Disease, *PR* Prevalence Ratio, *CI* Confidence Interval

### Association between SIBO and hepatic steatosis and Fibrosis in NAFLD

SIBO also not associated with significant hepatic steatosis (*p* = 0.951; PR = 0.951; CI 95% [0.452–2.239]) and fibrosis (*p* = 0.371; PR = 1.369; CI 95% [0.608–3.772]) (Tables 4 and 5 respectively)

**Table 4:** Bivariate Analysis Association between SIBO and Significant Steatosis

Variables	Significant Steatosis		Total (%)	*p* value	PR	CI 95%
	Yes	No				
SIBO, n (%)						
Yes	23 (63.9)	13 (36.1)	36 (100)	0.951	1.026	0.452–2.329
No	50 (63.3)	29 (36.7)	79 (100)			

*SIBO* Small Intestinal Bacterial Overgrowth, *PR* Prevalence Ratio, *CI* Confidence Interval

**Table 5:** Bivariate Analysis Association between SIBO and Fibrosis Degree

Variable	F0-F1	F2-F4	Total	*p* value	PR	CI 95%
SIBO, n (%)						
Yes	26 (72.2)	10 (27.8)	36 (100)	0.371	1.514	0.608–3.772
No	63 (79.7)	16 (20.3)	79 (100)			

*SIBO* Small Intestinal Bacterial Overgrowth, *PR* Prevalence Ratio, *CI* Confidence Interval

.

### Association between metabolic factors and SIBO

Metabolic factors such as DM, dyslipidemia, and obesity don’t have any strong association with the presence of SIBO, however, the presence of central obesity has significantly associated with the presence of SIBO (*p* = 0.001; PR = 0.378; CI 95% [0.021–0.478]) (Table 6).

**Table 6:** Bivariate Analysis Association between Comorbidities and SIBO

Variables	SIBO, n (%)		Total	*p* value	PR	CI 95%
	Yes	No				
DM, n (%)						
Yes	46 (38.3)	74 (61.7)	120 (100)	0.068	2.141	0.935–4.902
No	9 (22.5)	31 (77.5)	40 (100)			
Dyslipidemia, n (%)						
Yes	43 (33.3)	86 (66.7)	129 (100)	0.571	0.792	0.352–1.780
No	12 (38.7)	19 (61.3)	31 (100)			
Obesity, n (%)						
Yes	34 (31.8)	73 (68.2)	107 (100)	0.325	0.710	0.358–1.407
No	21 (39.6)	21 (60.4)	53 (100)			
Metabolic Syndrome, n (%)						
Yes	42 (34.4)	80 (65.6)	122 (100)	0.980	1.010	0.469–2.174
No	13 (34.2)	25 (65.8)	38 (100)			
Central Obesity, n (%)						
Yes	46 (30.9)	103 (69.1)	149 (100)	0.001	0.099	0.021–0.478
No	9 (81.8)	2 (18.2)	11 (100)			

*SIBO* Small Intestinal Bacterial Overgrowth, *DM* Diabetes Mellitus, *PR* Prevalence Ratio, *CI* Confidence Interval

### Gut Microbiome quantification in NAFLD

There are 60 patients with NAFLD who underwent gut microbiome quantification from the stool samples which includes Bifidobacteria (Genus of Actinobacteria), Lactobacillus (Genus of Firmicutes), and Bacteroides (Genus of Bacteroidetes). The quantification of Bifidobacteria and Lactobacillus didn’t show any statistically significant between non-significant hepatic fibrosis group and hepatic fibrosis group (p .880; p .222) (Table 7), however, there is a statistically significant of the quantification of Bacteroides in the significant fibrosis group when compared to the non-significant fibrosis group. The correlation using Spearmen test between the presence of Bacteroides and the stage of fibrosis also showed statistically significant (p .037) (Table 8). Further analysis between obese NAFLD patients and non-obese NAFLD patients showing that there is a statistically significant decrease of Bifidobacteria (p .047) and Lactobacillus (p .038) in obese NAFLD patients and a tendency of increase Bacteroides in obese NAFLD patients (p .572) (Table 9).

**Table 7:** Gut Microbiota quantification between non-significant hepatic fibrosis & significant hepatic fibrosis group. Quantity of bacteria (copy number DNA per gram fecal)

Species	All patients (*N* = 60)	Non Significance Fibrosis F0-F1 (*N* = 35)	Significance Fibrosis F2-F4 (*N* = 25)	*P* value
*Bifidobacteria*	6.79 × 10^3^ (4.25 × 10^4^)	1.05 × 10^4^ (5.56 × 10^4)^	1.61 × 10^3^ (4.67 × 10^3)^	0.880^c^
*Lactobacillus*	4.77 × 10^4^ (1.32 × 10^5^)	3.44 × 10^4^ (7.63 × 10^4^)	6.64 × 10^4^ (1.86 × 10^5^)	0.222^c^
*Bacteroides*	2.32 × 10^5^ (2.80 × 10^5^)	1.85 × 10^5^ (2.27 × 10^5^)	2.98 × 10^5^ (3.34 × 10^5^)	0.130^c^

^c^Man Whitney test

**Table 8:** Correlation between gut microbiota and stage fibrosis

Species	Spearmen correlation (r)	*P*-value
*Bifidobacteria*	0.057	0.667
*Lactobacillus*	0.102	0.436
*Bacteroides*	0.270	0.037

Spearman correlation

**Table 9:** Gut Microbiota quantification between NAFLD non-obese and NAFLD obese group. Quantity of bacteria (copy number DNA per gram fecal)

Species	All patients (*N* = 60)	Non Obese (*N* = 32)	Obese (*N* = 28)	*P* value
*Bifidobacteria*	6.79 × 10^3^ (4.25 × 10^4^)	1.30 × 10^4^ (6,22 × 10^4^)	1.36 × 10^3^ (4.20 × 10^3^)	.047
*Lactobacillus*	4.77 × 10^4^ (1.32 × 10^5^)	7.29 × 10^4^ (1.78 × 10^5^)	2.57 × 10^4^ (7.01 × 10^4)^	0.038
*Bacteroides*	2.32 × 10^5^ (2.80 × 10^5^)	2.08 × 10^5^ (2.73 × 10^5^)	2.53 × 10^5^ (2.89 × 10^5)^	0.572

Student’s t test

## Discussions

To our knowledge, this is the first comprehensive human study in Asia looking at the presence of SIBO and gut microbiota quantification in NAFLD patients and other metabolic factors with assessment of liver disease progression evaluated using CAP-TE.

Based on our study the prevalence of SIBO was higher in NAFLD patients when compared to non-NAFLD patients, however, there is no significant association between the presence of SIBO and NAFLD development. High prevalence of SIBO have been also reported from previous studies [10, 11]. Prevalence of SIBO in our NAFLD subjects (31.3%) is in line with the other studies in western countries and also quite similar to studies conducted in Iran (39%) [12] and India (37.5%) [13] as the Asian counterparts. This phenomenon is in part due to shifts of Asian people habit who nowadays have an easier access to consume western style diets [14–16] highs in calories, saturated fats, carbohydrate, artificial sweeteners and also high fructose.

Study by Sabate in 2008 revealed that SIBO was more prevalent in obese subjects and it is similar to our findings [17]. Obesity and metabolic syndrome have been considered as the strongest risk factors for NAFLD development [18, 19], and SIBO is one of an important factor for obesity development. Fermentation activity process of the gut microbiota will be increased from the excessive polysaccharide source of diet and lead to excessive short-chain fatty acid (SCFA) and monosaccharides production, where it can lead to the obesity condition based on acetate production. Obesity itself might alter intestinal permeability which can further increase the presence of SIBO due to bacterial translocation [20]. Excessive free fatty acids due to high-calorie or high-fat content food is one of important contributor to the NAFLD development [21].

There are major concerns about GHBT examination where it might be difficult to exclude the presence of SIBO when the result was negative, the sensitivity and specificity were reported only 40 and 80%, and it is also not possible to diagnose the presence of SIBO in the distal ileum, however, studying gut microbiota might hold a promise to overcome this matter in the future. Until now there is no standard cut off yet for duodenal or Jejunal aspiration examination which has been considered as the gold standard test. In fact, there is also no consensus yet about the gold standard test for detection the presence of SIBO. Another consideration about non-hydrogen producer people might be missed out through the GHBT examination [22]. GHBT is still a simple test and easy to use in clinical practice and SIBO is not the only factor that is important in NAFLD management.

Analysis on metabolic factors such as DM, dyslipidemia, central obesity, and metabolic syndrome were found not significant as risk factors for NAFLD development even though there are tendency to be higher prevalence in NAFLD population than in non-NAFLD population with metabolic risk factors. NAFLD is a complex and multi-factorial disease as it is strongly related to the insulin resistance condition. In our cohort patients, the presence of SIBO was not proofed as an independent risk factor for advanced liver fibrosis in our NAFLD patients. Liver fibrosis is still a part of dynamic process, and this was only a cross-sectional study design. However, the high prevalence of SIBO and other metabolic factors in NAFLD patients when compared to non-NAFLD patients have given an insight about possibility of more complex interplay (molecular and metabolic crosstalk) for disease progression in NAFLD as well as its impact on management. We further analyzed our data and interestingly found that the only metabolic factor which has significant association with SIBO in our cohort patients is central obesity. This finding might give further explanation since most of studies come from Western countries where there are many influence factors can be different in the presence of SIBO in Asian countries such as type of diet, body mass index (BMI), medications, and environment.

There are three phyla which have important role in the presence of SIBO and metabolic disorders, Firmicutes (Gram positive), Actinobacteria (Gram positive), and Bacteroidetes (Gram negative). The gut microbiota in adults mostly dominated by Firmicutes (60–80%) and Bacteroidetes (20–40%) [20]. Based on gut microbiota PCR analyses in our study showed there are significant tendency to be lower in amount of Bifidobacteria (genus of Actinobacteria) and Lactobacillus (genus of Firmicutes) in obese NAFLD patients when compared to non-obese NAFLD patients. Interestingly, the increase of Bacteroides (genus of Bacteroidetes) is statistically significant associated with the degree of fibrosis stage. This fecal quantification using RT-PCR of gut microbiota has strengthen our previous SIBO result using hydrogen breath test that the presence of SIBO is more related to the presence of obesity than the NAFLD itself. This result also showed a different result to the previous animal study which showed gut microbiota can contribute to the NAFLD development independently of obesity. However, some studies still showed conflicting results regarding the presence of SIBO in NASH and also the ratio of good and bad bacteria in NAFLD [20, 23].

There are some limitations of this study, first this study used a cross-sectional design which might not represents the dynamic process of the complex pathogenesis in NAFLD and could not reveal causal relationships between variables. However, NAFLD is still a complex disease and it is not easy to do comprehensive routine evaluation in daily practice. Second, the presence of SIBO is only evaluated by using glucose hydrogen breath test (GHBT) where it might not have covered the distal ileum bacterial overgrowth and also non-hydrogen producer. However, performing Jejunal aspirates examination is not easy as it can get easily contamination from the oral flora or saliva. At this moment, GHBT is still the most practical test to detect the presence of SIBO. Third, all of our patients didn’t undergo liver biopsy, however, despite its invasiveness and possibility of sampling error, TE-CAP has been validated and demonstrated satisfied AUROC [24–26]. Fourth, our study lacks nutritional assessment which may have differences in terms of daily food habit between eastern vs. western countries. Fifth, our study also lacks in assessing the effects of cigarette smoking as it may interfere the gut microbial composition.

## Conclusions

The presence of SIBO has no significant association in NAFLD development. SIBO plays an important role more in NAFLD patients with obesity.

## Abbreviations

BMI: Body Mass Index
CAP: Controlled Attenuation Parameter
DM: Diabetes Mellitus
GHBT: Glucose Hydrogen Breath Test
IBD: Inflammatory Bowel Disease
IBS: Irritable Bowel Syndrome
NAFLD: Non-Alcoholic Fatty Liver Disease
NASH: Non-Alcoholic Steatohepatitis
SCFA: Short Chain Fatty Acid
SIBO: Small Intestinal Bacterial Overgrowth
TE: Transient Elastography
